# Underestimated Cervical Cancer among Women over 65 Years Old: Is It Time to Revise the Screening Target Age Group?

**DOI:** 10.1055/s-0043-1772477

**Published:** 2023-12-23

**Authors:** Renata Alfena Zago, Deolino João Camilo-Júnior, Solange Correa Garcia Pires D'Ávilla, José Cândido Caldeira Xavier-Júnior

**Affiliations:** 1 School of Medicine, Centro Universitário Católico Unisalesiano Auxilium, Araçatuba, SP, Brazil; 2 Instituto de Patologia de Araçatuba, Araçatuba, SP, Brazil; 3 Faculdade de Medicina de São José do Rio Preto, São Paulo, SP, Brazil; 4 Faculdade de Medicina de Botucatu, Universidade Estadual Paulista, Botucatu, SP, Brazil

**Keywords:** aging health, cytology, papanicolaou test, screening, uterine cervical neoplasms, saúde do idoso, citologia, teste de papanicolau, programas de rastreamento, neoplasias do colo do útero

## Abstract

**Objective** To compare cytological and histological results from women > 64 years old who followed the Brazilian national cervical cancer screening guidelines with those who did not.

**Methods** The present observational retrospective study analyzed 207 abnormal cervical smear results from women > 64 years old in a mid-sized city in Brazil over 14 years. All results were reported according to the Bethesda System. The women were divided into those who followed the screening guidelines and those who did not.

**Results** Atypical squamous cells of undetermined significance and low-grade squamous intraepithelial lesion cytology results were found in 128 (62.2%) cases. Of these, 112 (87.5%) had repeated cytology with positive results. The other 79 (38.1%) with abnormal results should have been referred to colposcopy and biopsy. Out of 41 (51.9%) biopsied women, 23 (29.1%) had a confirmed diagnosis of neoplasia or precursor lesion. In contrast, among the 78 (37.7%) biopsied patients, 40 (51.3%) followed the guideline recommendations, with 9 (22.5%) positive biopsies. Of the 38 (48.7%) women who did not follow the guidelines, there were 24 (63.1%) positive results. Women who did not follow the guidelines demonstrated higher chances of cancer and precursor lesions (odds ratio [OR]: 5.904; 95% confidence interval [CI]: 2.188–15.932; *p* = 0.0002).

**Conclusion** Women > 64 years old who did not follow the national screening protocol showed significant differences in the frequency of abnormal results and severity of diagnosis compared with those who followed the protocol.

## Resumo

**Objetivo** Comparar os resultados citológicos e histológicos de mulheres > 64 anos que seguiram as diretrizes nacionais brasileiras de rastreamento do câncer do colo do útero com aquelas que não as seguiram.

**Método** O presente estudo observacional retrospectivo analisou 207 resultados anormais de esfregaço cervical de mulheres > 64 anos de idade em uma cidade de médio porte no Brasil durante 14 anos. Todos os resultados foram relatados de acordo com o Sistema Bethesda. As mulheres foram divididas entre as que seguiram as diretrizes de rastreamento e as que não o fizeram.

**Resultados** Resultados citológicos com células escamosas atípicas de significado indeterminado e lesão intraepitelial escamosa de baixo grau foram encontrados em 128 (62,2%) casos. Destes, 112 (87,5%) repetiram a citologia com resultados positivos. Os outros 79 (38,1%) com resultados anormais deveriam ter sido encaminhados para colposcopia e biópsia. Das 41 (51,9%) mulheres biopsiadas, 23 (29,1%) tiveram diagnóstico confirmado de neoplasia ou lesão precursora. Em contrapartida, entre as 78 (37,7%) pacientes biopsiadas, 40 (51,3%) seguiram as recomendações da diretriz, com 9 (22,5%) biópsias positivas. Entre as 38 (48,7%) mulheres que não seguiram as orientações, houve 24 (63,1%) resultados positivos. As mulheres que não seguiram as diretrizes demonstraram maiores chances de câncer e lesões precursoras (odds ratio [OR]: 5,904; intervalo de confiança [IC] de 95%: 2,188–15,932; *p* = 0,0002).

**Conclusão** Mulheres > 64 anos que não seguiram a diretriz nacional de rastreamento apresentaram diferenças significativas na frequência de resultados anormais e gravidade do diagnóstico em comparação com aquelas que seguiram a diretriz.

## Introduction

Despite national guidelines for screening and treatment, 1 cervical cancer (CC) is the fourth most deadly cancer in Brazilian women. 2 Worldwide, the incidence of CC in 2020 was 13.3 per 100,000 women, and the mortality was 7.3 per 100,000. 3 In Brazil, the mortality was 6.12 deaths per 100,000 women in 2022, 2 and among women > 65 years old (screened or not), it was 22.1% between 1996 and 2015. 4

The Brazilian CC screening program targets women aged 25 to 64 years old based on conventional cytology. 1 The first two tests should be performed yearly, and if both results are negative, the tests should be performed every 3 years. However, almost half of the tests occur within a year, 1 5 that is, some women were overscreened. In contrast, others are unscreened because all cytological tests are spontaneous; thus, only women who seek health services undergo cytologic examinations. 5

In this context, there are various explanations for the high incidence and mortality rates, including the low coverage rate of cytology, the opportunistic nature of the program, and the fact that there are no testing intervals or age group restrictions. 5 6 Also, there is almost no control over the amount or quality of the latest tests performed on older women who reach 64 years old when screening stops. 7 8 According to the guidelines, a patient should not reach the age limit without considering her screening history; it is critical to have at least two negative tests in the previous 5 years and no prior history of preinvasive neoplastic disease before ceasing cytological collections. 1 In this context, the present study compared the follow-up of cytological results from women > 64 years old and biopsied patients who did or did not adhere to the Brazilian national CC screening guidelines.

## Methods

The present observational, retrospective and analytical study compared the prevalence of abnormal cervical smears in women > 64 years old who did or did not follow the screening protocol. Our cohort came from Araçatuba, a mid-sized city in the southeastern countryside of the state of São Paulo, Brazil, and its region. The sample consisted of conventional cervical smears obtained from the records of the Instituto de Patologia de Araçatuba from January 1, 2002, to December 31, 2015 (14 years). This laboratory receives tests collected for CC screening from patients of the Brazilian Unified Health System (SUS, in the Portuguese acronym). Smears were collected from private clinics in Araçatuba and surrounding areas.

The results were reported according to the Bethesda System: atypical squamous cells of undetermined significance (ASC-US); atypical squamous cells cannot exclude high-grade squamous intraepithelial lesion (ASC-H); low-grade squamous intraepithelial lesion (LSIL); high-grade squamous intraepithelial lesion (HSIL); squamous cell carcinoma (SCC); atypical glandular cells of undetermined significance (AGC-US); atypical glandular cells favor neoplastic (AGC); endocervical carcinoma in situ; invasive cervical adenocarcinoma; invasive endometrial adenocarcinoma; and adenocarcinoma not otherwise specified. 9 There are additional categories in the Brazilian national guidelines: atypical undetermined cells of undetermined significance and atypical undetermined cells, which cannot exclude high-grade intraepithelial lesions. Both refer to results in which it is impossible to determine if the atypical cells are glandular or squamous. 1

Patients with abnormal results were compared in a subsequent step: repeat cytology in 6 months or go to colposcopy and biopsy, depending on the first abnormal cytology result. Then, those who were biopsied were divided into two groups: those who had at least two consecutive negative cytopathological tests in the previous 5 years (that is, those who followed the national CC screening guidelines and those who did not). 1 The magnitude of association was analyzed using the odds ratio (OR) with a 95% confidence interval (CI). Data were expressed as absolute ( *n* ) and relative (%) frequencies to assess the association between diagnostic categories. The significance level was set at 5%. Our research ethics committee approved the study under protocol CAAE: 83847517.10000.5379.

## Results

Over these 14 years, there were 207 abnormal cytological results among women > 64 years old. Of these, 120 (58.0%) were classified as ASC-US and 8 (3.9%) were LSIL. According to the national screening program, 1 these patients should undergo repeat cytology in 6 months: 112 (87.5%) repeated the cytology and only 33 (25.7%) showed an abnormal result in the second exam. Finally, 33 (25.7%) biopsies were performed in this group, of which 7 (5.5%) demonstrated some abnormality: 1 cervical intraepithelial neoplasia (CIN) I, 3 CIN II, 1 CIN III, 1 SCC and 1 endometrial carcinosarcoma. The other 79 (38.1%) patients with abnormal results should have been referred to colposcopy and biopsy; however, 43 (54.4%) repeated the cytology, with 22 abnormal results (1 ASC-US, 3 AGUS, 9 ASC-H, 1 AGCH, 1 LSIL, 5 HSIL, 1 SCC and 1 atypical undetermined cells that cannot exclude high-grade intraepithelial lesions). Biopsy was performed in 41 (51.9%) of the women in this group, and 23 (29.1%) were positive (2 endometrial adenocarcinomas, 9 SCC, 5 CIN II, 5 CIN III and 2 adenocarcinomas). The results of the first cytology and their follow-up are shown in Table 1 .

**Table 1:** The frequency of abnormal cytology and follow-up among women over 64 years old compared with following national guidelines

Cytologic results	First cytology	Repeated cytology	Abnormal results in second cytology	Biopsies	Abnormal biopsies
ASC-US	120 (58.3%)	105 (87.5%)	23 ASC-US 9 ASC-H 1 LSIL	29 (24.2%)	8 (6.6%)
ASC-H	28 (13.6%)	12 (42.8%)	6 ASC-H 2 HSIL	14 (50.0%)	8 (28.6%)
Atypical undetermined cells of undetermined significance	16 (7.8%)	12 (75%)	1ASCUC 1 AGUS 1 Atypical undetermined cells cannot exclude high-grade intraepithelial lesions	7 (43.7%)	2 (12.5%)
AGUS	9 (4.4%)	7 (7,8%)	1 AGCH 1 AGUS	3 (33.3%)	2 (22.2%)
LSIL	8 (3.9%)	7 (87.5%)	3 ASC-US	6 (75%)	1 (12.5%)
HSIL	8 (3.9%)	6 (75%)	2 ASC-H 3 HSIL	5 (62.5%)	2 (25%)
SCC	7 (3.4%)	2 (28.6%)	1 SCC 1 ASC-H	7 (100%)	6 (85.7%)
Atypical undetermined cells cannot exclude high-grade intraepithelial lesions	5 (2.4%)	3 (60%)	1 HSIL	3 (60%)	1 (20%)
AGC	3 (1.5%)	1 (33.3%)	1 AGUS	2 (66,7%)	1 (33.3%)
Adenocarcinoma not otherwise specified	1 (0.5%)	–	–	1 (100%)	1 (100%)
Invasive endometrial adenocarcinoma	1 (0.5%)	–	–	1 (100%)	1 (100%)
Invasive cervical adenocarcinoma	1 (0.5%)	–	–	–	–
Total	207 (100%)	155 (75.2%)	56 (27.1%)	78 (37.7%)	33 (15.9%)

Abbreviations: AGC: atypical glandular cells favor neoplastic; AGC-US: atypical glandular cells of undetermined significance; ASC-H: atypical squamous cells cannot exclude HSIL; ASC-US: atypical squamous cells of undetermined significance; CI: confidence interval; HSIL: high-grade squamous intraepithelial lesion; SCC: squamous cell carcinomas; LSIL: low-grade squamous intraepithelial lesion.

*All frequencies are relative to the total of the first cytology

Over the entire period, 78 (37.7% of the first abnormal results) biopsies were performed. Of these, 40 (51.3%) had at least 2 negative tests consecutively in the previous 5 years, following the national protocol: 31 (77.5%) negative biopsies and 9 (22.5%) positive results (1 endometrial adenocarcinoma, 1 SCC, 2 CIN I, 2 CIN II, 3 CIN III). Among the 38 (48.8%) women who did not follow the guidelines, 14 (36.8%) biopsies were negative and 24 (63.1%) were positive (1 endometrial carcinosarcoma, 1 endometrial carcinoma, 9 squamous cell carcinomas, 2 cervical adenocarcinomas, 5 CIN II, and 6 CIN III). The biopsy results are shown in Table 2 . Then, women who did not follow the guidelines demonstrated higher chances of cancer and precursor lesions (OR: 5.904; 95%CI: 2.188–15.932 *;* *p* = 0.0002).

**Table 2:** Frequency of abnormal biopsies among women over 64 years old compared with following national guidelines

Histological subtypes	Followed guideline	Not followed guideline	OR (95% CI)	Total
Negative	31 (77.5%)	14 (36.8%)	1	45 (57.7%)
Premalignant lesions (CIN I, II, and III)	7 (17.5%)	11 (28.9%)	3.479 (1.114–10.864)	18 (23.1%)
SCC	1 (2.5%)	9 (23.7%)	NA	10 (12.8%)
					Cervical adenocarcinoma	–	2 (5.3%)	NA	2 (2.6%)
Others	1 (2.5%, endometrial adenocarcinoma)	2 (5.3%, endometrial carcinosarcoma and endometrial adenocarcinoma)	NA	3 (3.8%)					
					Total	40	38	5.904 (2.188–15.932) p = 0.0002	78 (100%)

Abbreviations: CI: confidence interval; CIN: cervical intraepithelial neoplasia; OR: odds ratio; SCC: squamous cell carcinoma.

## Discussion

There is a significant frequency of CC precursor lesions and neoplasm in Brazilian women > 64 years old. A recent screening history influences the frequency and severity of the abnormal diagnosis. Many women in this age group with abnormal cytology did not correctly follow the screening protocols to confirm or treat the abnormality.

When women had indications to repeat the cytology because of their low-grade characteristics, 87.5% of the women did so. Under the Bethesda System, ASC-US suggests LSIL (CINI); however, with a 10 to 20% possibility of HSIL (CIN II or CIN III). 9 The Brazilian guidelines assume this degree of benign behavior of the alteration and make conservative recommendations; thus, women with ASC-US and LSIL cytology results should undergo repeat cytology in 6 months. 1

It is essential to highlight that some degree of neoplasia or premalignant lesions was found in 5.5% of biopsied patients. Other studies showed that conventional cytology had an overall sensitivity of 50 to 75% for detecting low-grade lesions and of 55 to 90% for high-grade lesions (CIN II/III). 8 10 11

Considering women whose cytological results have high-grade characteristics with indications to proceed directly to colposcopy and possible biopsy, 54.4% underwent a second cytological test, not following the current guidelines. Of this group, 29.1% had some type of neoplasm in a later biopsy. This finding suggests an underestimated number of CC diagnoses and a higher accumulated risk of CC in women who did not undergo screening as recommended, primarily among those with a high-grade lesion possibility. 12 13 In India, the frequency of abnormal biopsies in women > 65 years old was also high (47.3%), demonstrating a higher frequency of cervical alterations among older women who continue the screening, corroborating the present study. 14

When analyzing biopsies from 15 women with premalignant lesions, only 18.7% had followed the guidelines; among 10 cases of SCC, only 1 had followed the protocols, although all cases of adenocarcinomas had improper screening history. For glandular lesions, the difficulty in representing endocervical cells, especially among older women with some grade of retraction, may explain the screening not being performed appropriately. 1 8 9 The Brazilian guidelines consider any atypical glandular cell high-risk and associated with CIN II/III or cancer. 1

Inadequate cervical screening in older women is a possible reason for delayed diagnosis and poor prognosis. 15 On the other hand, adequate screening can reduce the incidence of cervical cancer by 75%, as well as mortality. 16 Therefore, in agreement with the present study, women > 64 years old with inadequate screening had a higher risk of CC and worse outcomes. 17 18

Other studies showed that few women who reached the age of exiting screening programs had been adequately screened during the preceding years. 19 20 Indeed, among women in the target group, there was poor follow-up, low frequency, and precarious cellular representation in samples, which may lead to underestimation of the prevalence of CC and premalignant lesions during screening of women at the target age. 21 In the context of inadequate cervical screening program performance, the frequency of cervical cancer could be more significant than expected.

In countries that implemented screening using DNA testing, high-risk human papillomavirus was present in smears of women > 70 years old, and there were premalignant lesions in 45% of them even after their exit from screening. 22 This finding indicates the importance of screening these women later in life, especially if they had an abnormal screening history or were not screened. These findings reinforce the relevance of reassessing the age of exit of the protocol, the quality of smears and the frequency of previous screening.

We identified (63.1%) severe abnormalities in older women who did not follow the guidelines (endometrial carcinosarcoma, SCC, adenocarcinomas, CIN II and CIN III). This finding is similar to the American scenario, where lesions in advanced stages may be explained by irregular screening history despite the guidelines. 23 The decreasing interest in screening with advancing age also explains why older women have higher incidences of CC, especially where screening programs have an opportunistic character, as is the case in Brazil. 24 25 These explanations were also advanced in Australia 26 and Finland 27 to explain the frequency of abnormal tests in older women with a history of inadequate screening.

The suboptimal screening performance among this group can be explained by the level of patient education regarding the disease and limited access to the test. 28 The lack of knowledge of health professionals in Brazil (and worldwide) about the target ages and subsequent steps in national protocols for diagnosing, monitoring, and treating precursor lesions and neoplasm can also explain the results. 29 30

A limitation of the present study is that we analyzed data from a medium-sized city, which might not represent all Brazilian populations. Nevertheless, the present study illustrates the prevalence of abnormal cervical smear results in our community since our laboratory is the only pathology laboratory in the city.

## Conclusion

Because CC mortality in Brazil is high, the frequency of abnormal cytological results among women > 64 years old is not insignificant. The present study demonstrated that women who did not follow the national guidelines had higher rates of true precursor lesions (CIN II/III) and invasive neoplasms (SCC, adenocarcinomas, and others) than those who followed the guidelines. These findings suggest revising the screening exit age in Brazil to reduce the incidence of CC.
